# Practical evaluation of the R.E.N.A.L. score system in 150 laparoscopic nephron sparing surgeries

**DOI:** 10.1590/S1677-5538.IBJU.2021.0424

**Published:** 2021-09-10

**Authors:** Victor T. Dubeux, José Fernando C. Zanier, Pedro N. Gabrich, Fabricio B. Carrerette, José C. A. Milfont, Ronaldo Damião

**Affiliations:** 1 Universidade do Estado do Rio de Janeiro - UERJ Departamento de Urologia Rio de Janeiro RJ Brasil Departamento de Urologia, Universidade do Estado do Rio de Janeiro - UERJ, Rio de Janeiro, RJ, Brasil

**Keywords:** Laparoscopy, Proof of Concept Study, Surgical Procedures, Operative

## Abstract

**Introduction:**

Nephrometric scores play an interesting role in nephron sparring surgery (NSS) planning. The aim of this study is to evaluate if R.E.N.A.L. score (RS) is capable to predict the occurrence of adverse events in laparoscopic NSS.

**Materials and Methods:**

We prospectively studied 150 laparoscopic NSS between 2015 and 2018 to evaluate the relationship between RS and incidence of adverse events. Clavien 3 or superior complications, warm ischemia time (WIT) over 30 minutes, tumor violation, positive surgical margins (PSM) and necessity of amplification of renal parenchyma during the resection of the masses to obtain free margins were considered as adverse events. We compared each item of the RS isolated and divided the patients between low risk and high risk.

**Results:**

Adverse results occurred in 48 cases (32%). Amplification of the margin of resection was observed in 28 cases (19%). WIT exceeded 30 minutes in 9 cases (6.1%), complications Clavien 3 or superior occurred in 13 cases (9%) and PSM were detected en 6 cases (4%). Comparing the patients with adverse outcomes and each item of the RS we did not find any statistical difference, but when divided into high risk and low risk, we found that patients in the high risk group had a higher tendency to present ad-verse results - 25.84% vs. 44.26% (p=0.03).

**Conclusions:**

RS system is a good way to predict adverse outcomes in NSS, especially in cases over 7. Further studies should focus on robotic approach and patient's characteristics other than the masses’ aspects.

## INTRODUCTION

There has been observed a shift towards performing nephron sparring surgeries (NSS) for low stage renal lesions in the last decades (1). This has happened especially due to wide access to radiological exams, leading to early diagnosis of those lesions, and as a natural consequence, NSS emerged as an oncologically equivalent alternative to radical nephrectomy in most cases of localized renal masses, and even in higher stage lesions (1, 2).

Conventional open NSS has been for the last 20 years the established standard option for removal of T1 tumors, but, minimally invasive approach has gained popularity over open NSS owing to its advantages of less blood loss, reduced operation time, shorter hospital stay, and avoidance of morbidity related to flank incisions (3, 4). However, laparoscopic NSS has a higher complication rate when compared with open access, and it is also reported to be demanding and technically challenging specially for complex renal tumors (3, 5).

In the absence of level one evidence favoring any of these techniques, recommendations are subject of biases such as surgeon's experience, and comfort levels (6). A lesion that one clinician may consider aggressive or even impossible for NSS, due to its location or size may be suitable for another surgeon, according to the team expertise and experience.

In order to establish the risk of adverse results in minimally invasive NSS, such as prolonged warm ischemia time, bleeding, tumor violation, and postoperative complications, several scoring systems have been proposed. The most widely reported are the C-index, the PADUA score and R.E.N.A.L. nephrometry (7, 8). Several groups have reported the use of these scoring systems in predicting perioperative outcomes and incidence of complications (9, 10).

Proposed by Kutikov et al. in 2009 (11), the R.E.N.A.L. score (RS) has been utilized in many situations for predicting the outcome in NSS. This scoring system is based on the radiological characteristics of the lesion, involving size, parenchymal depth, proximity to the colleting system and location related to the kidney itself. According with the radiological aspect, each item may vary from one to three points, and at the end those points are added to stylish the final score. Suffix h is applied if the tumor is located at the renal hilum, and letters P, A and X if the location is posterior, anterior or impossible to be determined.

We proposed a prospective evaluation of the utility of the RS in predicting the occurrence of adverse results and postoperative complications in the universe of laparoscopic NSS. To our knowledge, this study is the first to prospectively use the RS system in a large Brazilian cohort undergoing laparoscopic NSS in a tertiary referral center.

## MATERIALS AND METHODS

We prospectively evaluated 148 consecutive patients treated with NSS for renal masses (solid and cystic) suspected for malignancy. After approval of our ethics commission, a total of 150 renal masses were treated between March 2015 and Mach 2018, since two patients presented with two renal masses in the same kidney.

The same radiologist, to determine the RS of each lesion, reviewed preoperative magnetic resonance (MRI) or computed tomography (CT). Multiphasic CT was performed by using a renal mass protocol, consisting of a non-enhanced data acquisition and data acquisition during the nephrographic (delay, 90 seconds) and excretory (delay 3-5 minutes) phases after the application of a 150mL of iodinated contrast agent at a constant flow rate of 3.5mL/sec. and using a 64-channel multidetectory CT scanner (Brilliance CT, Philips). MRI examinations were performed with a 1.5T machine using a body matrix phased-array coil (Optima MR360, GE Medical Systems). DW imaging was performed and ADC maps were generated.

In addition to DW imaging, patients underwent the following routine imaging sequences: multiplanar T2- and T1-weighted imaging before and after administration of contrast material in the arterial and venous phases. One dose of gadobutrol (0.1mmol per kilogram of body weight) was administrated. After contrast material injection, images were acquired during the corticomedullary (20 seconds), nephrogenic (70 seconds) and excretory (3minutes) phases.

**Figure 1:**
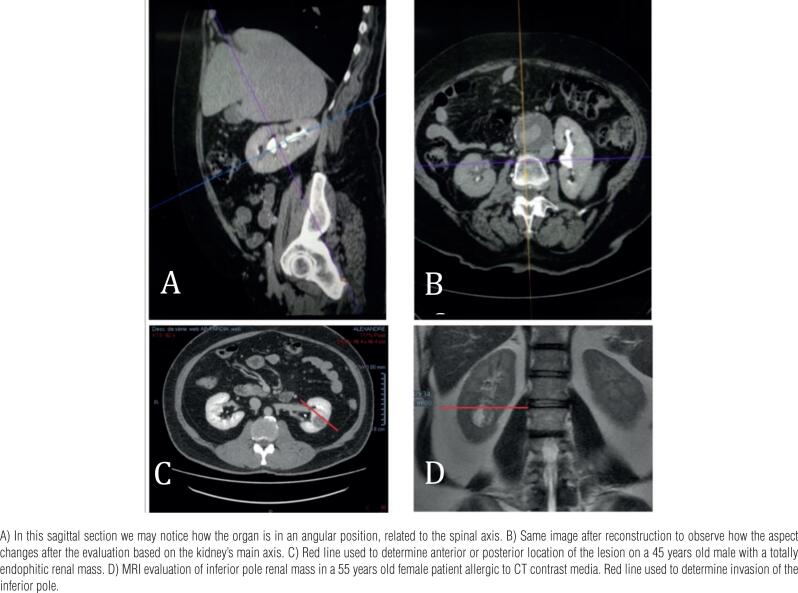
Kidney reconstruction based on the main renal axis. A – B a 65 years old male with an abdominal aneurism.

RS was determined based on images according to the renal main axis (Figure-1). Usually reconstructed images are done using the patient's spine as the main axis, and since the kidney is often presented in an angular position, it may miss the accurate position of the masses (Figure-1). The surgeons had access to the images, but not to the radiologist's review. All patients included in the study were submitted to pure laparoscopic NSS executed by three experienced surgeons (more than 100 procedures performed each), and signed an informed consent previously to the procedure.

Warm ischemia time (WIT) over 30 minutes, Clavien-Dindo (12) complications over 3 recorded in the first 90 days following the procedure, positive margins, tumor violation during resection and/or necessity of amplification of the area to be removed during NSS were considered as negative outcomes. The incidence of these negative outcomes was compared with RS in two ways. First - Each item of the R.E.N.A.L. score was compared with each case. So radius; exo-endofictness; nearness of the collecting system; location and relation to the polar lines were compared as independent characteristics to adverse outcomes. Second - According to the sum of the R.E.N.A.L. score, the cases were classified into high risk (RS over 7) and low risk (RS under 6) for NSS, to determine if the sum of the RS system was capable of predicting those adverse results.

## SURGICAL TECHNIQUE

**Figure 2:**
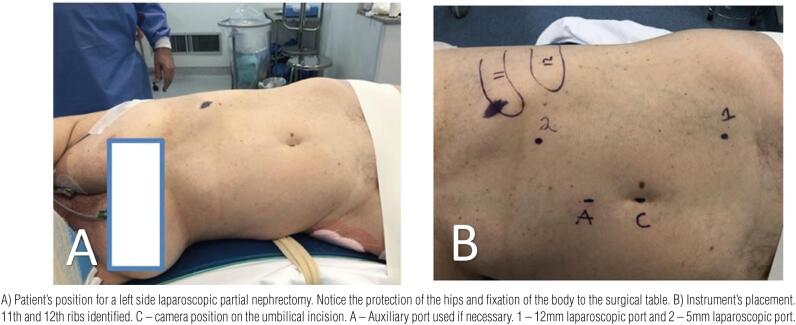
Patient's position for a left side partial nephrectomy.

All procedures were performed transperitoneally with the patients under modified lombotomy position. A camera port was inserted at the umbilicus, added to more three laparoscopic ports, two located at the hemiclavicular line and one at the tip of the 12^th^ rib (Figure-2). The procedure started with medial mobilization of the bowel, exposing the renal hilum, identification of the renal artery and vein. In lower pole lesions, the ureter was also identified to avoid lesions. The renal fat was removed exposing the lesion to be treated, and renal clamping was performed with laparoscopic bull dogs, according to the surgeon's preference either en bloc - artery and vein, artery only, and even without vascular clamping in some cases. After resected the lesion, the renorraphy was performed in two layers, one at the medullary region, closing the colleting system if necessary, and a second continuous suture to close the defect. The renal hilum was unclamped after the suture of the medullary region was performed, and the resected area was reevaluated for residual bleeding, and another layer of suture closing the edges of the resected area anchored by laparoscopic clips was performed. No hemostatic agents were utilized. Hemostasis was obtained using Gerota's fat and suture lines. The specimens were removed in proper laparoscopic bags, through the umbilicus, and a drain was installed in all cases. The patients started oral liquids as soon as the bowel movements returned, and were discharged from the hospital when drainage was insignificant.

## STATISTICAL ANALYSIS

A sample size of 150 masses was deemed sufficient to identify a Spearman's RHO of at least 0.25 between RS and adverse outcomes, with a 0.95 confidence interval and a statistical power of 0.8. We applied Fisher's exact T test and Pearson Chi square test to compare proportions and to establish possible correlation between RS and our cases, comparing the ones with adverse outcomes and their RS previously measured. A significance level of 0.05 was adopted for all statistical tests; data set were processed on Stata version 14.0 (2015. StataCorp. College Station, TX USA).

## RESULTS

We can observe the patients and renal masses aspects in Table-1.

**Table 1:** Patients’ distribution according to age, gender, body composition, ASA preoperative risk, pathological stage and subtypes.

DATA		
**Age**		62 (52-68)
**BMI**		22.77 (25.4 -31.14)
**Gender**		
	Female	64 (42.5%)
	Male	84 (57.5%)
**ASA**		
	I	55 (37.9%)
	II	85 (58.6%)
	III	5 (3.4%)
**Side**		
	Left	71 (53.4%)
	Right	62 (46.6%)
**Solid / Cystic**		
	Solid	128 (86.5%)
	Cystic	22 (13.5%)
**Pathological Stage**		
	T1a	98 (78.4%)
	T1b	19 (15.2%)
	T2	5 (4%)
	T3	3 (2.4%)
**Histological Subtype**		
	Clear Cells	124 (82.6%)
	Other malignant	1 (0.7%)
	Others benign	25 (16.7%)

Our median operative time has 120 min (90 - 130min), WIT was 19.5 min (9 - 39 min), and we have found 67 adverse results in 48 cases (32%). We established 30 minutes for WIT as an adverse event due to the controversy around this matter in the literature (13, 14) and it was detected in 9 cases (6.1%), 5 cases were RS 6, and in two cases each for RS 10 and 7. The collecting system was violated in 32 cases (21.8%), 15 cases were close to the collecting system (grade 3 in nearness) and in only 4 cases a urinary fistula was detected. Two cases were RS 7 and two RS 9. All four cases of urinary fistula were treated with double J stenting and a bladder catheter until the drainage stopped.

Amplification of the margin of resection was necessary in 28 cases (19.7%). RS 7 in 10 cases, RS 6 and 8 in 4 cases each, RS 5 in 3 patients, RS 10 in 2, RS 4 in 3, and one case in RS 11 and 9. Positive surgical margins on final pathology were detected in 6 cases (4.3%) two in RS 8, and RS 7, one in RS 9 and one in RS 5. Tumor violation was observed in 12 patients. In all cases, the margin of resection was amplified, to obtain normal parenchyma in the specimen. This occurred in RS 7 - 4 patients, RS 8 - 5 patients, RS 6 - 9 and 10, one patient each.

In a median period of follow-up of 3.5 (5–3) years, only one patient died due to metastatic disease, and in this specific case, there were no local recurrences.

Per operative complications were: Pleural perforation 1, bowel accidental perforation - 3, vascular injuries, 4 patients (1 Vena cava, 2 renal vein, 1 artery), all managed laparoscopically. Four patients had per operative bleeding over 1.0L, one was RS 4, two RS 5 and one in RS 6, all required monitoring in intensive care units. Two patients had their procedures converted to radical nephrectomy, one due to technical difficulty (equipment malfunction) and one due to hilar invasion of the lesion, making it impossible to preserve the organ (RS 11).

Postoperative complications detected were urinary fistula in 4 patients as reported previously, bleeding due to pseudoaneurism formation (one case RS 4), and bleeding requiring monitoring in intensive care unit in 2 cases (RS 8). Infections were detected in 4 patients (pneumonia, phlebitis, wound infection and urinary infection). One patient had to be submitted to a total nephrectomy due to acute bleeding on postoperative day 1 (RS 9).

Among the 89 patients with RS under 6, we have found adverse results in 23 (25.84%) and in the 61 patients with RS over 7, the incidence of adverse results was found in 27 cases (44.26%) p <0.05. The distribution of those patients is listed in Table-2 and Table-3 shows the incidence of adverse events according to each item of the RS and when the cases are divided in to low and high-risk groups.

**Table 2:** Incidence of adverse events according to the final R.E.N.A.L. score.

R.E.N.A.L.	4	5	6	7	8	9	10	11
# patients	28 (18.7%)	31 (20.7%)	30 (20%)	35 (23.3%)	13 (8.7%)	6 (4%)	5 (3.3%)	2 (1.3%)
WIT >30	0	0	5	2	0	0	2	0
Complications	3	2	2	2	0	2	1	1
Margin Amplification	3	3	4	10	4	1	2	1
Positive Margins	0	1	0	2	2	1	0	0
Tumor Violation	0	0	1	4	5	1	0	0
**Total**	**6**	**6**	**12**	**20**	**11**	**5**	**5**	**2**

**Table 3:** Patient's distribution according to each item of the renal score and divided between low and high risk.

R.E.N.A.L.		No Adverse Results	Adverse Results	Total Cases	P Value
**RADIUS**					Pearson Chi2-0.454
					Fisher's - 0.350
	1 (<4cm)	71 (70.3%)	30 (29.7%)	101	
	2 (4-7cm)	24 (58.54%)	17 (41.46%)	41	
	3 (>7cm)	5 (62.5%)	3 (37.5%)	8	
**EXOFITIC/ENDOFITIC**					Pearson Chi2-0.454
					Fisher's - 0.424
	1 (>50% exof.)	63 (65.62%)	33 (34.38%)	96	
	2 (50% exof.)	33 (71.74%)	13 (28.26%)	46	
	3 (<50% exof.)	4 (50%)	4 (50%)	8	
**NEARNESS**					Pearson Chi2 - 0.07
					Fisher's - 0.07
	1 (>7mm)	59 (78.67%)	16 (21.33%)	75	
	2 (4-7mm)	16 (51.61%)	15 (48.39%)	31	
	3 (< 4mm)	25 (56.82%)	19 (43.18%)	44	
**ANTERIOR/ POSTERIOR**					Pearson Chi2 -0.095
					Fischer's - 0.072
	A - Anterior	61 (75.31%)	20 (24.69%)	81	
	P - Posterior	26 (55.32%)	21 (44.68%)	47	
	H - Hilar	1 (100%)	0 (0.0%)	1	
	x - Undetermined	12 (60%)	8 (40%)	20	
**LINES**					Pearson Chi2 -0.322
					Fischer's - 0.324
	1 - Beyond Polar Lines	62 (70.45%)	26 (29.55%)	88	
	2 - Crossing Polar Lines	25 (65.79%)	13 (34.21%)	38	
	3 - Between Polar Lines	13 (54.17%)	11 (45.83%)	24	
**GROUPS ACCORDING TO RISK**					
	R.E.N.A.L. <6	66 (74.16%)	23 (25.84%)	89	
	R.E.N.A.L. >7	34 (55.74%)	27 (44.26%)	61	
					Pearson Chi 2 0.019
					Fisher's – 0.03

## DISCUSSION

Size limit for CT1 NSS has shifted from 4cm to 7cm in size and it also may be indicated in some CT2 cases when feasible (2, 15, 16). With the development of minimally invasive techniques, the possibility to estimate the risk of occurrence of an adverse outcome is of great importance when indicating this approach, specially in high complexity cases. The target of an ideal NSS should be a good oncological outcome with negative surgical margins, maximal renal function preservation and minimized complications. Those goals are achieved with low warm ischemia time, low bleeding, no tumor violation and good hemostasis (17, 18).

In this scenario, clinical decision-making is subjective, since there are aspects including not only the tumor characteristics, but also institutional experience, surgical team expertise and patient's related factors, such as age, comorbidities, amount of fat evolving the kidney and anatomic issues among others that should be taken in consideration when indicating a minimally invasive NSS (19).

Despite subjective factors involving decision making, there are some aspects related specifically to the lesion itself that of most importance. Specially in those patients, nephrometry scores play a very important role.

This detailed description should include not only the adequate location of the lesion on the kidney surface, specially in those small tumors, that are sometimes difficult to locate, but also the depth of penetration of the mass through the parenchyma. The deepest you dissect the tissue, the higher are the chances of reaching larger blood vessels and consequently bleeding complications tend to occur. Invasion of the collecting system, and relation to the polar lines are also important aspects to be described, since they may be related to other adverse outcomes, such as urinary fistulas or loss of renal parenchyma due to suture hemostasis (20).

An important issue evolving nephrometry is the inter observer variability and the lack of standardization of the radiological evaluation and the absence of inclusion of aspects not related to the tumor (21). In our series, the determination of the RS was stablished using the main renal axis, which minimized the inter observer differences. Another important aspect is that most series evaluating RS are retrospective, what makes it difficult to evaluate specifically some aspects due to confounding factors (22). In this series, we previously stablished which were the technical difficulties in a laparoscopic NSS, such as tumor violation and indication on margin amplification, and also determined the adverse results to be studied before evaluating the postoperative results.

Since RENAL nephrometry system adresses all these charctaristics related to the masses, it has made it the most popular and widely used (1). But still, this system has some issues to be considered. If we compare two tumors with similar scores it does not mean that they do necessarily pose the same characteristics. For an exemple, a 3cm left sided anterior exophitic tumor (RENAL 4a) in a healthy slim individual might be much easier to ressect than a 2cm upper pole right posterior mass in a diabetic obese patient (RENAL 4p).

That brings us the main question. Is RS score system a way to predict surgical outcome or simply a more detailed way to describe a renal mass? In our series, we evaluated it in two ways. First, each item of the renal score isolated, then by dividing the patients into high and low risk groups.

When considering each item isolated we were not able to predict adverse outcomes (Table-3), but when added to one another and stratified by high and low risk, a statistically significant result was obtained, suggesting that when the sum of RS is equal or over 7 (61 masses out of 150 total), there is a higher incidence or occurrence of an adverse outcome (44.26% vs. 25.84%) (Table-2).

Our incidence of adverse events is comparable to series described in the literature with an incidence of 4.3% of positive surgical margins, with no impact in overall survival in a short follow-up time. Usually, relevant factors related to local recurrence are high Fuhrman grade and stage T2-T3a (21–23).

Tumor violation is another relevant aspect of NSS. In our 12 cases when this happened in a short period of follow-up, we did not detect any cases of recurrence, probably due to low aggressive neoplasia or no spillage of malignant cells as reported by other authors (24, 25). We observed in our data regarding tumor violation, positive margins and necessity of amplification of the area of resection that RS wasn't able to predict, since that was observed in high (18 cases) and low risk patients (10 cases), with a higher tendency to occur in RS scores over 7.

We stablished a 30 minutes warm ischemia time as a cut off for an adverse result due to the lack of information in the literature concerning a specific period of time that a kidney should have its blood flow safely interrupted with no damage to the renal function (26, 27). All of our 9 patients with warm ischemia time over 30 minutes were discharged home with a serum creatinine level close to the preoperative levels. Interestingly, five were low risk (RS 5) and four high risk (2 RS 10 and 2 RS 7). Certainly, if we considered a lower WIT time, like in some papers published (13, 14, 22), we would have more cases with adverse outcomes. Interestingly, a long WIT was necessary in five cases of low risk and four for the high risk group (Table-3). This is opposite to what we see in other papers, with a higher WIT for the cases with high complexity tumors (28, 29).

A higher complication rate with an incidence of Clavien 3 or superior incidence, was also previously described for the high risk group of patients submitted to NSS (29, 30); this was not observed in our series, since among all 13 cases when it happened, seven were in the low risk group (RS <6).

Another issue that should be adressed in nephrometry scores is the patient's characteristics. Aspects such as the amount of fat around the kidney (31), anatomic considerations, number of arteries, previous abdominal procedures, comorbidities and preoperative renal function are relevant factors and may impact on the final result of a NSS, and are not taken under consideration in any nephrometry score system.

Recently, robotic surgery has gained acceptance among minimally invasive urologic surgeons due to several advantages, such as three-dimensional image magnification, dexterous movements and short learning curve. Some reports show that robotic assisted NSS is safe and feasible with good short term oncological outcome, and possibility to be performed in complex cases with better results. Further experience is needed to validate RS in this scenario (32, 33). Laparoscopy is still performed in a lot of countries where the access to robotic surgery is limited, even in complex cases (34). So, evaluating RS and laparoscopic surgery is usefull for these institutions when indicating a laparoscopic NSS or referring the patient to an institution with robotic surgery technology. Also, if robotic assisted surgery has better results when compared to pure laparoscopy, the incidence of adverse events in high complexity RS lesions should be lower, and the results of this study could be even better if evaluated on robotic assisted series.

## CONCLUSION

Laparoscopic NSS for small renal masses presenting with RS nephrometry system sum over 7 should be managed by experienced surgeons, since those cases might be associated with a higher incidence of complications and adverse results. Further studies should focus on robotic assisted approach and adding patient's characteristics other than the aspect of the renal mass isolated to increase accuracy in predicting adverse events.

## ABBREVIATIONS

NSS: nephron sparring surgery
RS: R.E.N.A.L. score nephrometry system
PSM: positive surgical margins
WIT: warm ischemia time
BMI: body mass index
ASA: classification of physical status of the “American Society of Anesthesiologists”

## References

[1] Uzzo RG, Novick AC. Nephron sparing surgery for renal tumors: indications, techniques and outcomes. J Urol. 2001; 166:6-18. 11435813

[2] Deng H, Fan Y, Yuan F, Wang L, Hong Z, Zhan J, et al. Partial nephrectomy provides equivalent oncologic outcomes and better renal function preservation than radical nephrectomy for pathological T3a renal cell carcinoma: A meta-analysis. Int Braz J Urol. 2021; 47:46-60. 10.1590/S1677-5538.IBJU.2020.0167 PMC7712695 32271510

[3] Wang Y, Ma X, Huang Q, Du Q, Gong H, Shang J, et al. Comparison of robot-assisted and laparoscopic partial nephrectomy for complex renal tumours with a RENAL nephrometry score ≥7: peri-operative and oncological outcomes. BJU Int. 2016; 117:126-30. 10.1111/bju.13214 26132424

[4] Aron M, Haber GP, Gill IS. Laparoscopic partial nephrectomy. BJU Int. 2007; 99(5 Pt B):1258-63. 10.1111/j.1464-410X.2007.06815.x 17441920

[5] Dominguez-Escrig JL, Vasdev N, O’Riordon A, Soomro N. Laparoscopic partial nephrectomy: Technical considerations and an update. J Minim Access Surg. 2011; 7:205-21. 10.4103/0972-9941.85643 PMC3193690 22022109

[6] Campbell-Walsh Urology, 9th Edition (Edition) - Edited by AJ Wein, LR Kavoussi, AC Novick, AW Partin and CA Peters. 2007. Vol. 1, pp.144-57.

[7] Ficarra V, Novara G, Secco S, Macchi V, Porzionato A, De Caro R, et al. Preoperative aspects and dimensions used for an anatomical (PADUA) classification of renal tumours in patients who are candidates for nephron-sparing surgery. Eur Urol. 2009; 56:786-93. 10.1016/j.eururo.2009.07.040 19665284

[8] Simmons MN, Ching CB, Samplaski MK, Park CH, Gill IS. Kidney tumor location measurement using the C index method. J Urol. 2010; 183:1708-13. 10.1016/j.juro.2010.01.005 20299047

[9] Hew MN, Baseskioglu B, Barwari K, Axwijk PH, Can C, Horenblas S, et al. Critical appraisal of the PADUA classification and assessment of the R.E.N.A.L. nephrometry score in patients undergoing partial nephrectomy. J Urol. 2011; 186:42-6. 10.1016/j.juro.2011.03.020 21571340

[10] Bruner B, Breau RH, Lohse CM, Leibovich BC, Blute ML. Renal nephrometry score is associated with urine leak after partial nephrectomy. BJU Int. 2011; 108:67-72. 10.1111/j.1464-410X.2010.09837.x 21087391

[11] Kutikov A, Uzzo RG. The R.E.N.A.L. nephrometry score: a comprehensive standardized system for quantitating renal tumor size, location and depth. J Urol. 2009; 182:844-53. 10.1016/j.juro.2009.05.035 19616235

[12] Dindo D, Demartines N, Clavien PA. Classification of surgical complications: a new proposal with evaluation in a cohort of 6336 patients and results of a survey. Ann Surg. 2004; 240:205-13. 10.1097/01.sla.0000133083.54934.ae PMC1360123 15273542

[13] Marberger M. Renal ischaemia: not a problem in laparoscopic partial nephrectomy? BJU Int. 2007; 99:3-4. 10.1111/j.1464-410X.2007.06612.x 17227485

[14] Funahashi Y, Hattori R, Yamamoto T, Kamihira O, Kato K, Gotoh M. Ischemic renal damage after nephron-sparing surgery in patients with normal contralateral kidney. Eur Urol. 2009; 55:209-15. 10.1016/j.eururo.2008.07.048 18706758

[15] Ljungberg B, Bensalah K, Canfield S, Dabestani S, Hofmann F, Hora M, et al. EAU guidelines on renal cell carcinoma: 2014 update. Eur Urol. 2015; 67:913-24. 10.1016/j.eururo.2015.01.005 25616710

[16] Long CJ, Canter DJ, Kutikov A, Li T, Simhan J, Smaldone M, et al. Partial nephrectomy for renal masses ≥7 cm: technical, oncological and functional outcomes. BJU Int. 2012; 109:1450-6. 10.1111/j.1464-410X.2011.10608.x 22221502

[17] Basu S, Khan IA, Das RK, Dey RK, Khan D, Agarwal V. RENAL nephrometry score: Predicting perioperative outcomes following open partial nephrectomy. Urol Ann. 2019; 11:187-92. 10.4103/UA.UA_93_18 PMC6476220 31040606

[18] Alvim RG, Audenet F, Vertosick EA, Sjoberg DD, Touijer KA. Performance Prediction for Surgical Outcomes in Partial Nephrectomy Using Nephrometry Scores: A Comparison of Arterial Based Complexity (ABC), RENAL, and PADUA Systems. Eur Urol Oncol. 2018; 1:428-34. 10.1016/j.euo.2018.05.004 PMC9199452 31158083

[19] Bai N, Qi M, Shan D, Liu S, Na T, Chen L. Trifecta achievement in patients undergoing partial nephrectomy: a systematic review and meta-analysis of predictive factors. Int Braz J Urol. 2021; 47. Epub ahead of print. 10.1590/S1677-5538.IBJU.2021.0095 PMC9306373 34115456

[20] Parsons RB, Canter D, Kutikov A, Uzzo RG. RENAL nephrometry scoring system: the radiologist's perspective. AJR Am J Roentgenol. 2012; 199:W355-9. 10.2214/AJR.11.8355 22915426

[21] Shah PH, Moreira DM, Okhunov Z, Patel VR, Chopra S, Razmaria AA, et al. Positive Surgical Margins Increase Risk of Recurrence after Partial Nephrectomy for High Risk Renal Tumors. J Urol. 2016; 196:327-34. 10.1016/j.juro.2016.02.075 PMC9235535 26907508

[22] Matos AC, Dall’Oglio MF, Colombo JR Jr, Crippa A, Juveniz JAQ, Argolo FC. Predicting outcomes in partial nephrectomy: is the renal score useful? Int Braz J Urol. 2017; 43:422-31. 10.1590/S1677-5538.IBJU.2016.0315 PMC5462132 28266814

[23] Ani I, Finelli A, Alibhai SM, Timilshina N, Fleshner N, Abouassaly R. Prevalence and impact on survival of positive surgical margins in partial nephrectomy for renal cell carcinoma: a population-based study. BJU Int. 2013; 111:E300-5. 10.1111/j.1464-410X.2012.11675.x 23305148

[24] Breda A, Stepanian SV, Liao J, Lam JS, Guazzoni G, Stifelman M, et al. Positive margins in laparoscopic partial nephrectomy in 855 cases: a multi-institutional survey from the United States and Europe. J Urol. 2007; 178:47-50. 10.1016/j.juro.2007.03.045 17574057

[25] Yossepowitch O, Thompson RH, Leibovich BC, Eggener SE, Pettus JA, Kwon ED, et al. Positive surgical margins at partial nephrectomy: predictors and oncological outcomes. J Urol. 2008; 179:2158-63. 10.1016/j.juro.2008.01.100 PMC2704565 18423758

[26] Funahashi Y, Hattori R, Yamamoto T, Kamihira O, Kato K, Gotoh M. Ischemic renal damage after nephron-sparing surgery in patients with normal contralateral kidney. Eur Urol. 2009; 55:209-15. 10.1016/j.eururo.2008.07.048 18706758

[27] Thompson RH, Frank I, Lohse CM, Saad IR, Fergany A, Zincke H, et al. The impact of ischemia time during open nephron sparing surgery on solitary kidneys: a multi-institutional study. J Urol. 2007; 177:471-6. 10.1016/j.juro.2006.09.036 17222613

[28] Hayn MH, Schwaab T, Underwood W, Kim HL. RENAL nephrometry score predicts surgical outcomes of laparoscopic partial nephrectomy. BJU Int. 2011; 108:876-81. 10.1111/j.1464-410X.2010.09940.x 21166761

[29] Simmons MN, Ching CB, Samplaski MK, Park CH, Gill IS. Kidney tumor location measurement using the C index method. J Urol. 2010; 183:1708-13. 10.1016/j.juro.2010.01.005 20299047

[30] Turna B, Frota R, Kamoi K, Lin YC, Aron M, Desai MM, et al. Risk factor analysis of postoperative complications in laparoscopic partial nephrectomy. J Urol. 2008; 179:1289-94. 10.1016/j.juro.2007.11.070 18289584

[31] Davidiuk AJ, Parker AS, Thomas CS, Leibovich BC, Castle EP, Heckman MG, et al. Mayo adhesive probability score: an accurate image-based scoring system to predict adherent perinephric fat in partial nephrectomy. Eur Urol. 2014; 66:1165-71. 10.1016/j.eururo.2014.08.054 25192968

[32] Aboumarzouk OM, Stein RJ, Eyraud R, Haber GP, Chlosta PL, Somani BK, et al. Robotic versus laparoscopic partial nephrectomy: a systematic review and meta-analysis. Eur Urol. 2012; 62:1023-33. 10.1016/j.eururo.2012.06.038 22771266

[33] Castilho TML, Lemos GC, Cha JD, Colombo JR, Claros OR, Lemos MB, et al. Transition from open partial nephrectomy directly to robotic surgery: experience of a single surgeon to achieve “TRIFECTA”. Int Braz J Urol. 2020; 46:814-21. 10.1590/S1677-5538.IBJU.2019.0101 PMC7822359 32648421

[34] Bauza JL, Tubau V, Guimerà J, Ladaria L, Aliaga C, Piza P, et al. Retroperitoneoscopic approach for highly complex posterior renal hilar tumors. Int Braz J Urol. 2020; 46:485-6. 10.1590/S1677-5538.IBJU.2019.0074 PMC7088471 32167727

